# Influence of Processing Temperature on Membrane Performance and Characteristics of Process Streams Generated during Ultrafiltration of Skim Milk

**DOI:** 10.3390/foods9111721

**Published:** 2020-11-23

**Authors:** Ritika Puri, Upendra Singh, James A. O’Mahony

**Affiliations:** 1 School of Food and Nutritional Sciences, University College Cork, T12 TP07 Cork, Ireland; sa.omahony@ucc.ie; 2 Lakeland Dairies, Bailieborough, A82 N6K8 Co. Cavan, Ireland; singhu@lakeland.ie

**Keywords:** ultrafiltration, milk, temperature, retentate, permeate, viscosity, plasmin

## Abstract

The effects of processing temperature on filtration performance and characteristics of retentates and permeates produced during ultrafiltration (UF) of skim milk at 5, 20, and 50 °C were investigated. The results indicate that despite higher flux at 50 °C, UF under these conditions resulted in greater fouling and rapid flux decline in comparison with 5 and 20 °C. The average casein micelle diameter was higher in retentate produced at 5 and 20 °C. The retentate analysed at 5 °C displayed higher viscosity and shear thinning behaviour as compared to retentate analysed at 20 and 50 °C. Greater permeation of calcium and phosphorus was observed at 5 and 20 °C in comparison with 50 °C, which was attributed to the inverse relationship between temperature and solubility of colloidal calcium phosphate. Permeation of α-lactalbumin was observed at all processing temperatures, with permeation of β-lactoglobulin also evident during UF at 50 °C. All UF retentates were shown to have plasmin activity, while lower activity was measured in retentate produced at 5 °C. The findings revealed that UF processing temperature influences the physicochemical, rheological, and biochemical properties of, and thereby govern the resulting quality and functionality of, retentate- and permeate-based dairy ingredients.

## 1. Introduction

Ultrafiltration (UF) has a wide range of applications in dairy processing, such as protein standardisation of cheese milk, production of liquid protein concentrates, high protein powders and novel milk products such as protein-enriched milk [1]. The choice of processing temperature strongly influences the entire UF process, and consequently, the structure and functionality of protein ingredients derived therefrom [2]. Traditionally, UF was most commonly conducted at high temperature, ≈50 °C; however, low temperature UF at less than 15 °C has become more widely adopted by the dairy industry in recent years [3,4,5]. It is reported that low temperatures can control microbial growth during filtration, restricting the increase in microbial load in resultant retentates [3]. Moreover, the rise in demand for more natural and less processed products has paved the way for high-quality dairy protein ingredients for various premium food applications including clinical, sports, and infant nutrition. In the USA, the legal temperature requirement for on-farm UF of milk is <7 °C, with the most common applications being production of whole UF milk and pre-concentration of cheese milk [6].

A commonly used criterion to evaluate membrane filtration performance is to measure the flux, which is generally higher for high temperature UF (≈50 °C), because of lower feed and permeate viscosity. However, such high temperatures can also give rise to undesirable microbial growth in produced retentates [4]. In addition, elevated temperature increases the probability of conformational changes to β-lactoglobulin (β-LG) dimers, which then convert into monomers, increasing thiol group reactivity, thereby promoting denaturation and aggregation [7]. In contrast, at low temperature (5–10 °C), microbial growth is significantly hindered but higher permeate viscosity results in lower permeate flux [8]. In addition, low temperature also increases the solubility of colloidal calcium phosphate and promotes the reversible dissociation of β-casein from casein micelles [9]. Apart from its impact on retentates, processing temperature can also modulate composition and physicochemical properties of permeate by influencing the permeation of minerals and proteins through the membrane. Overall, these temperature-induced changes can directly influence membrane performance by governing the fouling rate and cleaning efficiencies of the membrane [5], as well as affecting the functionality of generated retentates [10].

For instance, applying UF to skim milk at specific processing temperatures can impact the activity of plasmin, an indigenous milk proteinase that is technologically significant for many dairy products and high protein dairy ingredients due to its ability to hydrolyse all caseins, except κ-casein [11]. The activity of plasmin in milk can significantly increase if plasminogen, an inactive zymogen, is converted into plasmin by plasminogen activator [12]. Both plasmin and plasminogen are associated with casein micelles and are highly heat stable. The plasmin system also contains heat labile inhibitors, plasmin inhibitor, and plasminogen activator inhibitor, which exist in milk serum [13]. The UF of skim milk may influence the partitioning of these plasmin components on the basis of their permeation through the membrane. Moreover, the temperature-dependent activity of each plasmin component, particularly the action of plasminogen activator on inactive plasminogen, converting it into plasmin [11], could be influenced during UF of skim milk at specific processing temperatures. These membrane- and temperature-dependent changes in plasmin components have the ability to govern resulting plasmin activity in UF retentates. However, there is limited information available on the influence of membrane partitioning and processing temperature on plasmin activity in milk protein ingredients, either in fresh UF retentate or high protein powder [14].

Much of the research that has been conducted has focused on studying UF performance and associated changes in skim milk, at traditionally used high temperature (50 °C), with very limited work published on applying UF at lower temperatures. Given the importance of processing temperature in filtration, this study aimed to generate new scientific information in relation to UF of skim milk across a wider range of processing temperatures, more specifically its impact on UF operation, partitioning of milk constituents, and controlling technologically significant properties of retentates and permeates. Therefore, in this study on UF of skim milk, the influence of three processing temperatures, 5, 20, and 50 °C, was evaluated on filtration performance by measuring permeate flux and membrane permeability, before and after membrane cleaning. Besides studying filtration performance, characterising retentates and permeates would provide additional scientific and technical knowledge required to realise their potential as dairy ingredients in various food applications, particularly UF permeate, which was recognised as a food ingredient by Codex Alimentarius in 2017. Therefore, the properties of generated UF retentates and permeates were investigated using extensive characterisation in terms of composition, physicochemical properties, protein profile, and enzyme activity.

## 2. Materials and Methods

### 2.1. Materials

Low-heat skim milk powder was provided by a local Irish dairy company. Reconstituted skim milk powder was prepared by slow addition of powder to deionised water under constant magnetic stirring at 22 ± 1 °C. Once all powder was added, solutions were left stirring for 2 h and then stored overnight at 4 °C. A lactose assay kit was purchased from Abcam, Cambridge, UK. Urokinase enzyme (U4010) and N-succinyl-ala-phe-lys 7-amido-4-methyl (S0763) was purchased from Sigma-Aldrich, Arklow, Wicklow, Ireland.

### 2.2. Ultrafiltration of Skim Milk

Ultrafiltration (UF) experiments were performed as described by Crowley et al. [8]. A lab-scale pressure-driven cross-flow filtration rig and Biomax UF membrane cassette, 10 kDa, enclosed within a stainless steel membrane holder (Pellicon 2 mini-holder) was procured from Merck-Millipore (Tullagreen, Carrigtwohill, Co., Cork, Ireland). The new membrane was cleaned using instructions provided by the membrane manufacturer. In brief, the new membrane was initially flushed with ultrapure water to remove storage solution, followed by water recirculation for 20 min. The next step was to flush the membrane with 0.1–0.3 N NaOH solution, followed by NaOH recirculation for 50 min. After cleaning with NaOH, post cleaning flush and 20 min recirculation cycle was performed with ultrapure water. The entire cleaning process was performed at 50 °C. Normalized water permeability (NWP) of clean water was measured for a clean new membrane at transmembrane pressure (TMP) of 0.35 bar at 26 °C. This initial NWP of 221 (L/m^2^/h/bar) was used as a benchmark against which subsequent water permeability measurements were compared before and after cleaning the membrane on completion of each UF run. The continuously stirring feed (2.5 L) was delivered to the filtration unit using a peristaltic feed pump (model 520S, IP31, Watson-Marlow Fluid Technology Group, Watson-Marlow Limited, Ireland). In order to understand the relationship between permeate flux and transmembrane pressure (TMP) for operation at 5, 20, and 50 °C, we carried out a flux excursion exercise before concentration experiments at each processing temperature in full recirculation mode by returning the retentate and permeate lines back to the feed vessel. The feed flow rate was kept constant during each experiment to achieve the cross flow velocity of 0.34 m/s, recommended by Crowley et al. [8]. The TMP was increased stepwise in 0.1 bar increments by partially closing the valve on the retentate side. At each TMP increment, the feed flow was equilibrated for 3–4 min, which was confirmed to be acceptable for this lab filtration rig. After equilibration of feed flow, permeate flux was recorded in duplicate by measuring the volume of permeate.

For concentration experiments, TMP between 0.45–0.50 bar was selected as initial pressure to operate in the sub-critical flux region. The feed temperature was maintained throughout processing at 5 ± 0.5 °C, 20 ± 0.5 °C, and 50 ± 0.5 °C by recirculating retentate back into the feed vessel. The retentate line passed through a plate heat exchanger that received water in alternate channels supplied by a water bath set at the specific processing temperature. The processing temperature, TMP, and filtration duration was monitored and measured at regular intervals of 1, 1.5, 2, 2.5, 3, and 3.5 × volume concentration factor (VCF). The Brix value was measured at each interval using a portable refractometer (N1-a, 0e32% Brix, Atago USA Inc., Bellevue, WA, USA) [15]. Feed volume was reduced to 3.5 × VCF, where VCF was calculated as starting feed volume divided by final retentate volume. After each experiment, the membrane and filtration rig were cleaned thoroughly by flushing and recirculation with water and NaOH at 50 °C, as described above.

### 2.3. Characterisation of Process Streams

#### 2.3.1. Composition of Feed, Retentate, and Permeate Samples

The total protein content was determined using the Kjeldahl method with a nitrogen-to-protein conversion factor of 6.38 [16]. Ash content was determined by dry ashing in a muffle furnace at 500 °C for 5 h [8]. Total solids were measured by oven drying at 103 °C for 5 h [17]. The lactose content was determined using a lactose assay kit [3]. The pH was measured immediately after UF using a pH meter (bench pH meter, model HI2211-02, Hanna Instruments Ltd., Bedfordshire, UK) at 22 ± 1 °C. The ionic calcium (Ca^2+^) concentration was determined using a Ca ion-selective electrode at 25 °C, with a calibration curve constructed from 5 Ca^2+^ standard solutions (2, 4, 6, 8, and 10 mM) [8]. The instrument was calibrated with these standard solutions before each analysis. Conductivity was measured at 25 °C, with a 5-ring conductivity measuring cell (Metrohm Ireland Ltd., Athy Road, Co., Carlow, Ireland). Mineral profile was measured using inductively coupled plasma mass spectrometry [18].

#### 2.3.2. Viscosity and Particle Size Distribution of Feed and Retentate Samples

The viscosity measurement temperature was the same as the processing temperature applied during UF of skim milk. The viscosity of feed and fresh retentate samples was measured within 2 h at 5, 20, and 50 °C with a HAAKE RotoVisco 1 viscometer (Thermo Scientific, Dieselstrasse 4, D-76227, Karlsruhe, Germany) using a cup and bob geometry [15]. The viscosity was measured using a shear rate ramp from 10–1000 s^−1^. The data were fitted to the power law model to calculate consistency coefficient (K) and flow behaviour index (*n*), as described by Mun et al. [19]. The particle size distribution of feed and retentate samples was also measured within 2 h of processing by dynamic light scattering using a Zetasizer Nano series HT (Malvern Instruments Ltd., Worcestershire, UK) equipped with a He–Ne laser emitting at 633 nm and accompanying Malvern Zetasizer software v.7.02 [4]; parameters were set at a dispersant refractive index (RI) of 1.330 and viscosity of 0.8872 (cP). The dispersions were measured in disposable cuvettes and the material was characterised as protein with a RI of 1.45. The measurements were carried out at 25 °C after 120 s temperature equilibration using a backscattering configuration of 173°. Data were expressed as z-average, on the basis of intensity-weighted mean size of all particles in dispersions. Three independent samples from each processing temperature were analysed in duplicate. A total of 3 measurements were taken for each individual replicate. For analysis, feed solution was diluted 1:100, and retentate was diluted 1:1000, in tempered de-ionised water and immediately introduced into the instrument for measurement in order to maintain the integrity of casein micelles [20].

#### 2.3.3. Protein Profile Analysis

Protein profile of feed, retentate, and permeate streams was qualitatively assessed using sodium dodecyl sulphate–polyacrylamide gel electrophoresis (SDS-PAGE) with precast gels (Mini-PROTEAN TGX, Bio-Rad Laboratories, Irvine, CA, USA) under reducing conditions using an AcquaTank mini gel unit (Acquascience, Bellbrook Industrial Estate, Uckfield, UK) [2]. The final protein concentration of 1 mg/mL for feed and retentate samples was used for loading, and permeate was used as is. The sample solution of 8 μL was loaded for feed and retentate samples, while 20 μL was used for permeate samples, and all gels were Coomassie-stained. SDS-PAGE gels were scanned using a desktop scanner (HP Scanjet G4010, HP, Leixlip, Ireland) and densitometry was performed using GelAnalyzer 19.1 (www.gelanalyzer.com) by Istvan Lazar Jr., PhD and Istvan Lazar Sr., PhD, CSc.

#### 2.3.4. Plasmin and Plasminogen-Derived Activity

Plasmin and plasminogen-derived activity after activation of plasminogen by urokinase were determined in fresh feed, retentate, and permeate samples within 16 h from UF experiments by measuring the concentration of the fluorescent product 7-Amino-4-Methylcoumarin (AMC) released by plasmin from the fluorescent substrate, N-succinyl-l-alanyl-l-phenylalanyl-l-lysyl-7-amido-4-methyl coumarin. Plasmin and plasminogen assays were carried out as described by Richardson et al. [21]. Samples were diluted with 0.4 M trisodium citrate (TSC) at a 3:1 ratio before measurements. Samples were incubated with 50 mM Tris-HCl buffer (pH 7.5) at 22 °C for 10–15 min. For converting plasminogen into active plasmin, 500 µL of TSC-treated sample was incubated with 500 µL of urokinase solution (270 U/mL) for 60 min at 37 °C [22]. The reaction was initiated in final mixture by adding substrate, and the rate of peptide hydrolysis was determined by measuring the fluorescence of released AMC during incubation, at every 1 min up to 60 min. Standard curves were prepared by plotting the fluorescence intensity versus concentration of AMC (up to 600 nM) to calculate the rate of release of AMC. Plasminogen-derived activity was calculated by subtracting innate plasmin activity from total plasmin activity after activation of plasminogen by urokinase. The rate of increase in fluorescence intensity during the assay is proportional to the quantity of plasmin- or plasminogen-derived activity present. Plasmin activity was expressed in AMC units/g solids (nanomoles of AMC released per min) of sample.

### 2.4. Statistical Analysis

All results shown in this study for UF of skim milk, sample composition, physicochemical properties, protein profile, and plasmin- and plasminogen-derived activity are raw mean values analysed by fixed-effects one-way ANOVA. The data represent at least duplicate analysis of samples from 3 independent runs at each processing temperature. Means were tested for statistical significance (*p* < 0.05) by applying Duncan’s post-hoc test, using the software IBM SPSS Statistics 20.0 (IBM Corp. Released 2011. IBM SPSS Statistics for Windows, Version 20.0. Armonk, NY, USA).

## 3. Results and Discussion

### 3.1. Influence of Processing Temperature on Membrane Performance

**Figure 1:**
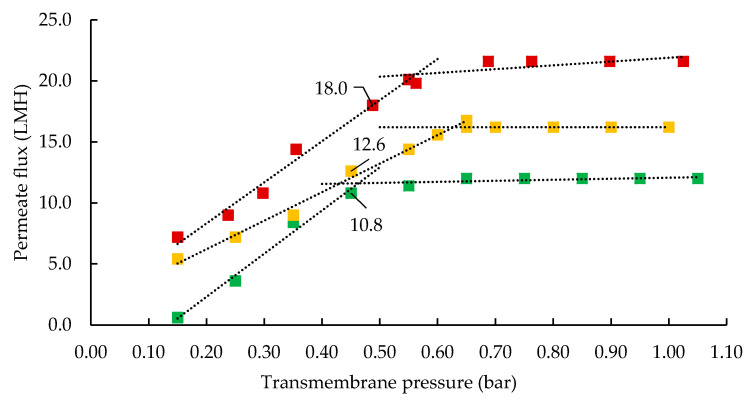
Flux excursion plot showing permeate flux as a function of transmembrane pressure (TMP) during ultrafiltration of skim milk in full recirculation mode with 10 kDa membrane at 5 ± 0.5 (■), 20 ± 0.5 (■), and 50 ± 0.5 °C (■). To study temperature as a single test variable, an initial TMP of 0.45–0.5 bar was selected as initial TMP to operate ultrafiltration (UF) experiments near the sub-critical flux region. Cross flow velocity was kept constant at 0.34 m/s. LMH: L/m^2^/h.

The relationship between permeate flux and transmembrane pressure (TMP) at 5, 20, and 50 °C is shown in Figure 1. The first part of these TMP excursion profiles is the pressure-dependent region, where flux increased with increasing TMP at each processing temperature. The flux was higher at 50 °C than 5 or 20 °C at any given TMP, most likely because of reduced feed and permeate viscosity at higher temperature [5]. The region of the flux–TMP curve where flux was largely independent of TMP is the pressure-independent region. This is due to accumulation of retained particles, including casein micelles and whey proteins on the membrane surface, generating resistance to fluid flow, which directly obstructs the permeate flow [23]. As flux started to plateau in the transition zone, at the knee of the flux excursion curve, the optimum TMP, ranging between 0.45 and 0.5 bar, was selected as the initial TMP to run UF concentration experiments [8]. A cross flow velocity of 0.34 m/s was selected to maintain turbulence around the membrane in order to increase wall shear stress by way of applying tangential force to the membrane [8]. This tangential movement intensifies flux by accelerating the removal of solutes from the membrane surface and also reduces the hydraulic resistance generated by deposited solutes [1].

**Figure 2:**
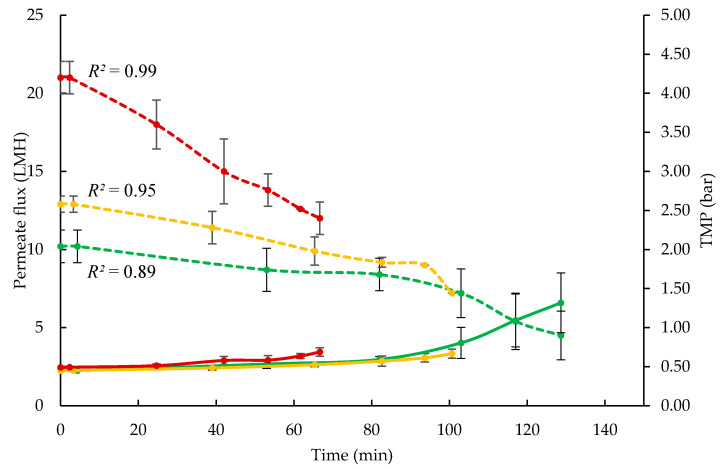
Permeate flux and transmembrane pressure (TMP) as a function of processing time during ultrafiltration of skim milk with 10 kDa membrane at 5 ± 0.5, 20 ± 0.5, and 50 ± 0.5 °C. Line colour represents flux at 5 °C (▪▪▪), 20 °C (▪▪▪), 50 °C (▪▪▪); TMP at 5 °C (▬), at 20 °C (▬), at 50 °C (▬). Values are raw mean ± standard deviation of data from triplicate runs at each test temperature. LMH: L/m^2^/h.

During UF processing, the permeate flux was measured at regular intervals, and it declined steadily with time at each processing temperature (Figure 2). The starting fluxes were 10.8, 12.6, and 18 L/m^2^/h (LMH) for feed at 5 °C (F5), 20 °C (F20), and 50 °C (F50), respectively. The *R*^2^ values of 0.89, 0.95, and 0.99, display the linearity of flux decline at 5, 20, and 50 °C, respectively, with the rate of flux decline being much more rapid at 50 °C than 5 or 20 °C. The brix value of feed increased from 9.5 to 23.3, 18.7, and 22.0, during UF concentration, at 5, 20, and 50 °C, respectively. The processing time required to reach 3.5 × feed volume reduction was 128, 100, and 60 min, at 5, 20, and 50 °C, respectively.

The flux decline observed in this study can be linked to a substantial role played by skim milk components (casein micelles, whey proteins, and minerals) in controlling the fouling mechanisms during UF. The particle deposition on, and pore blocking of, membranes can lead to fouling, resulting in gradual flux decline during UF [23]. The fouling mechanism, which consists of surface deposition and/or pore blocking, can take place through pore blocking, protein adsorption, mineral precipitation, or denaturation and aggregation of proteins on membrane surfaces [24]. For instance, as the smallest whey protein, α-lactalbumin (α-LA) can potentially penetrate through a 10 kDa UF membrane and result in pore blockage, causing membrane fouling [5], directly blocking pores and restricting permeate flux, as observed at all three processing temperatures in this study. The protein adsorption phenomenon, which generally takes place on nonporous areas of the membrane surface, could also have contributed to fouling during UF at 5, 20, and 50 °C [5]. Protein adsorption is a phenomenon in which the free energy of the system is minimised through (a) charge redistribution on protein surface, (b) release of surface adsorbed water and ions from parts of the protein and membrane, and (c) structural rearrangements of the protein molecule [23].

It has been reported that caseins have much lower driving forces for reduction in free energy and resulting adsorption on membrane surfaces than whey proteins, and therefore whey proteins dominate the adsorption on membrane surfaces [23]. Among major whey proteins, adsorption of α-LA is more extensive than β-LG, most likely due to accessibility of the smaller α-LA molecule to porous membrane surface in comparison with other proteins, which increases its contribution to protein adsorption-mediated fouling [24]. This phenomenon may explain the flux decline observed at all three processing temperatures. However, the rapid flux decline at 50 °C, as compared to steadier decline at 5 and 20 °C, could be linked to temperature-induced conformational changes in β-LG, from dimer to monomer, which takes place between 40 and 55 °C, at around neutral pH, also termed the Tanford transition [23]. These changes increase its hydrophobicity and expose its embedded thiol groups to thiol–disulphide interactions, which may lead to aggregation. This protein aggregation may support early onset of fouling, which could cause rapid flux decline, as observed at 50 °C. A similar observation were reported by Ng et al. [5], who conducted a foulant study of UF membrane using skim milk as feed material at 10, 30, and 50 °C, reporting more pronounced flux decline at 50 °C than 10 and 30 °C, contributed to by proteinaceous fouling material. Low molecular weight peptides and α-LA were most abundant at 10 and 30 °C, while at 50 °C, β-LG was also present in the foulant layer [5].

**Figure 3:**
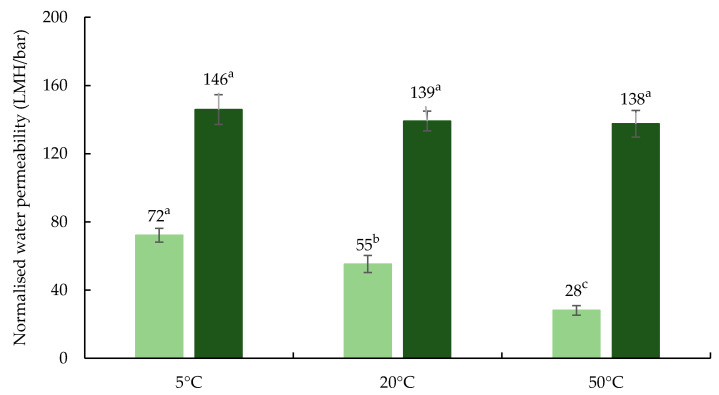
Normalised water permeability of membrane measured before (■) and after (■) membrane cleaning process. Values are the raw mean ± standard deviation of analysis from triplicate runs at each test temperature. Different superscripted lower-case alphabet letters within the bar graphs indicate significant differences (*p* < 0.05). LMH: L/m^2^h.

The membrane fouling due to mineral precipitation, particularly calcium phosphate, may also take place at all processing temperatures, especially within the membrane pores where casein and whey proteins are available to provide stability; however, during UF of skim milk, especially with organic membranes, the extent of this effect has been reported to be minor [5]. Moreover, precipitation of calcium in bulk fluid such as skim milk feed would not occur naturally, mostly due to its associations with casein micelles, its high buffering capacity, and its ability to establish equilibrium between colloidal and serum phase [3]. In addition, UF membrane molecular weight cut off was too large to retain minerals for enrichment, which may contribute to mineral precipitation [5]. Therefore, the extent of contribution of mineral fouling to flux decline during UF of skim milk at all three processing temperatures could be very limited. In the present study, the permeability of water through fouled membrane was measured before membrane cleaning, which was significantly (*p* < 0.05) lower at 50 °C than 5 and 20 °C (Figure 3), suggesting the restricted fluid flow through fouled membrane after UF at 50 °C, perhaps due to extensive pore blocking and layer formation by adsorbed proteins and to some extent mineral precipitation [23]. The NWP values of cleaned membrane measured after chemical cleaning ranged between 137 and 146 LMH/bar. This confirms the effectiveness of the cleaning regime followed each UF run in order to reach at least 60–70% of the benchmark NWP value, 221 LMH/bar, as recommended by the membrane manufacturer.

### 3.2. Composition of Retentate and Permeate Streams

The average content of total solids, protein, lactose, ash, and pH for feed, fresh retentate, and permeate produced at 5, 20, and 50 °C is shown in Table 1. The retentates denoted by R5, R20, and R50 maintained essentially the same pH as the original feed when measured at 22 ± 1 °C. The total solids content was 18.6% for R5, 15.4% for R20, and 20.4% for R50. The total protein content was 12.1% for R5, 9.8% for R20, and 13.7% for R50. The ionic calcium content measured at 25 ± 0.2 °C was 2.4 and 3 mM in retentate R5 and R20, respectively, which was significantly (*p* < 0.05) higher than 2.2 mM in R50. The mineral profile of retentates is shown in Table 2. Despite the higher ionic calcium content, the total calcium and phosphorus content in R5 and R20 were significantly (*p* < 0.05) lower than R50. This provides evidence for the (partial) solubilisation of colloidal calcium phosphate (CCP) within micelles as the temperature is lowered [3]. During UF, soluble minerals, including around 30% soluble calcium, are in diffusible forms and can permeate through the membrane [25]. Minerals derived from solubilised CCP as a result of low temperature may permeate through the membrane along with other innate soluble minerals [26,27]. This may explain the significantly (*p* < 0.05) higher values of total calcium and phosphorus in permeate, P5 and P20, as compared to P50. This is further supported by the significantly higher ionic calcium content in P5 and P20 than P50. The content of sodium, magnesium, potassium, and sulphur did not vary significantly in permeate samples, suggesting comparable permeation of these minerals at all three processing temperatures. The total protein content of P50 was significantly (*p* < 0.05) higher than P5. At high temperature, UF membranes tend to be slightly more open and permeable, which may lead to greater transmission of smaller, lower molecular weight proteins such as β-LG and α-LA, as observed in permeate P50 [7].

### 3.3. Physical Properties of Retentates

After each UF run, feed and retentate samples were analysed for casein micelle size distribution and zeta potential (Table 1) within 2 h of UF processing. The zeta potential of retentates did not vary significantly and showed comparable values of −26 mV. The average diameter of casein micelles in feed was comparable to retentate samples. The size of casein micelles was significantly (*p* < 0.05) smaller in retentate R50 at 179 nm, than R5 at 191 nm and R20 at 190 nm. During UF, temperature-dependent changes may take place within casein micelles and CCP of skim milk, which may consequently influence size and hydration of casein micelles [9]. At low temperature, mineral equilibrium shifts towards the soluble phase, as CCP partially solubilises [28]. As soluble minerals tend to permeate through the membrane during UF, this may have led to greater permeation of solubilised CCP constituents during UF at 5 and 20 °C. To establish mineral equilibrium between the colloidal and serum phases of the skim milk, further mineral displacement might have taken place at low temperature. Moreover, protein interactions within casein micelles are prone to modification at low temperature, as the β-casein tends to migrate from micellar to serum phase [29]. The overall mineral and protein shift from colloidal to serum phases may increase the hydration of casein micelles, resulting in swelling [30]. The relatively larger casein micelle size observed in retentate R5 and R20, may be associated with hydration and swelling of casein micelles in these retentates due to the temperature-mediated effects on mineral and casein protein equilibria. Liu et al. [9] studied the effect of temperature on casein micelle size by fractionating the casein micelles from skim milk using centrifugation and measuring micelle size by dynamic light scattering at 10, 20, and 40 °C. It was reported that the proportion of soluble casein decreased and the population of small casein micelles increased as the temperature increased from 10 to 40 °C; this may have been due to micellisation of soluble casein at high temperature due to increased strength of hydrophobic casein interactions.

**Figure 4:**
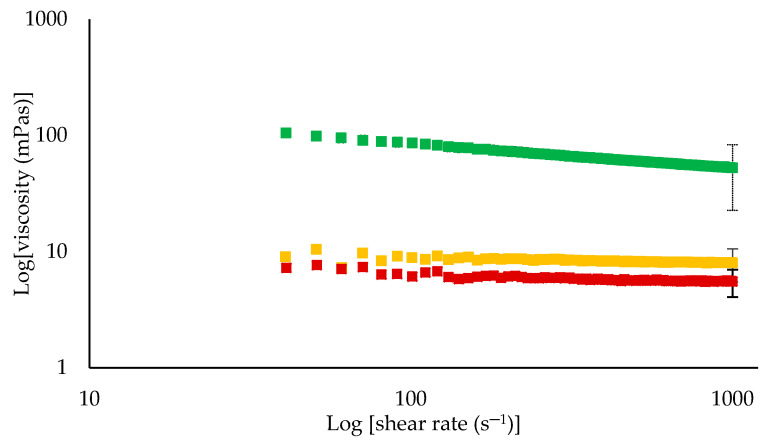
Log–log plots of viscosity as a function of shear rate for retentates obtained by ultrafiltration of skim milk at 5 ± 0.5 (■), 20 ± 0.5 (■), or 50 ± 0.5 °C (■). Values are raw mean ± standard deviation of duplicate analysis from triplicate runs at each test temperature.

The viscosity of feed and retentate samples was analysed at the same temperature used in UF processing. The application of the Power Law model to viscosity data revealed that all feed samples had a flow behaviour index (*n*) value of 1, indicating Newtonian liquid behaviour [19], while consistency coefficient (K) followed the order 0.003>0.002>0.001 for measurement at 5, 20, and 50 °C, respectively. The viscosity of feed increased during UF, with increasing protein content, regardless of processing temperature. However, the viscosity was significantly (*p* < 0.05) different between retentates, as shown by log–log plots of apparent viscosity and shear rate data in Figure 4. All retentates showed some degree of shear-thinning behaviour, as indicated by *n* <1.0; however, the extent of shear-thinning was more pronounced in R5 than R20 and R50. The K value was significantly (*p* < 0.05) higher for retentate R5 at 0.23 in comparison with R20 and R50, both with K values of 0.01. Concentrates containing less than 15% protein generally display Newtonian fluid behaviour [31]. At lower or moderate protein concentrations of less than ≈15%, the casein micelles behave as hard spheres, and as concentration of these hard spheres increases, the viscosity increases, as observed in all retentate samples, displaying higher K values than feed samples [31]. However, the higher viscosity of R5 can be linked to higher voluminosity of casein micelles at low temperature (≈5 °C) [32]. The casein micelle contains bound water inside and around its surface, which determines its voluminosity, which depends on temperature, pH, and ionic strength of the medium [32]. At low temperature, partitioning of minerals and the migration of β-casein from the inside to the colloidal surface and towards the serum phase causes the casein micelles to become more hydrated, resulting in increased voluminosity [9]. This increase in voluminosity is manifested as higher viscosity [15]. Sood et al. [33] reported that upon cooling skimmed human milk to 4 °C, the amount of protein associated with the micelles decreased and the volume of the casein micelle increased, suggesting increased casein micelle hydration as a causative factor. The lower consistency value of retentate R20 and R50 indicated strengthening of hydrophobic interactions between casein micelles, with higher processing temperature, which made micelles more compact, allowing free movement within colloidal suspension, resulting in lower viscosity [34].

### 3.4. Protein Profile of Retentate and Permeate Samples

**Figure 5:**
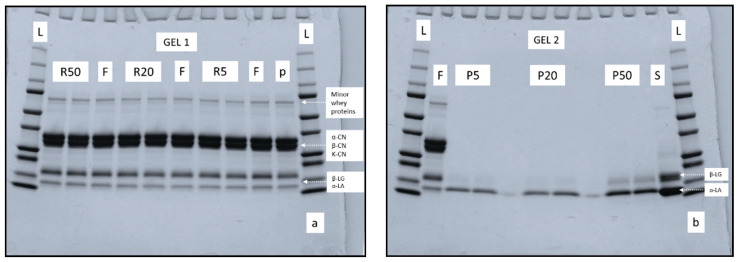
Electrophoretic patterns under reducing conditions of (**a**) retentate and (**b**) permeate samples obtained by ultrafiltration of skim milk at 5 ± 0.5, 20 ± 0.5, and 50 ± 0.5 °C. Bands represent samples from two independent runs at each processing temperature. L: ladder; F: feed; R: retentate; P: permeate; p: low-heat skim milk powder; S: α-LA standard; α-CN: α-casein; β-CN: β-casein; κ-CN: κ-casein; β-LG: β-lactoglobulin; α-LA: α-lactalbumin.

The protein profile of retentate and permeate samples produced at 5, 20, and 50 °C are shown in Figure 5a,b. According to densitometry analysis of bands, casein/whey, as well as α-LA/β-LG, were comparable for retentates R5 and R20, while both protein ratios were significantly (*p* < 0.05) higher in R50. The electrophoretic pattern of permeate samples revealed two protein bands in each permeate sample, generated at different processing temperatures, as shown in Figure 5b. The bands in each permeate sample corresponded to α-LA and β-LG when compared with migration distances of proteins in feed sample. According to densitometry analysis, the proportion of β-LG, in particular, was significantly (*p* < 0.05) higher in P50 as compared to P5 and P20, as also visible in corresponding intense bands shown in Figure 5b. At ≈50 °C and neutral pH, β-LG tends to undergo reversible conformational changes, (i.e., Tanford transition), resulting in conformational change from dimer to monomeric form [35]. These monomers of β-LG may permeate through the membrane during UF of skim milk, especially at higher processing temperatures, as the membrane tends to change to become more open, facilitating further permeation [7]. This may explain the higher permeation of β-LG in permeate P50 than in P5 and P20. [36]. The higher casein/whey and α-LA/β-LG ratios quantified in retentate R50 could also have been linked to this higher permeation of β-LG at 50 °C. A similar observation was made by Barbano et al. [37], who reported the presence of β-LG in UF permeates produced at 50 °C using a 10 kDa UF membrane. The presence of α-LA in all permeate samples, as revealed by electrophoretic pattern in Figure 5b, suggests the ability of α-LA, with physical dimensions of 2.3 nm × 2.6 nm × 4.0 nm [38], to permeate through 10 kDa UF membrane with mean pore diameter of around 5.1 nm [24]. 

### 3.5. Plasmin- and Plasminogen-Derived Activity in Retentates

**Figure 6:**
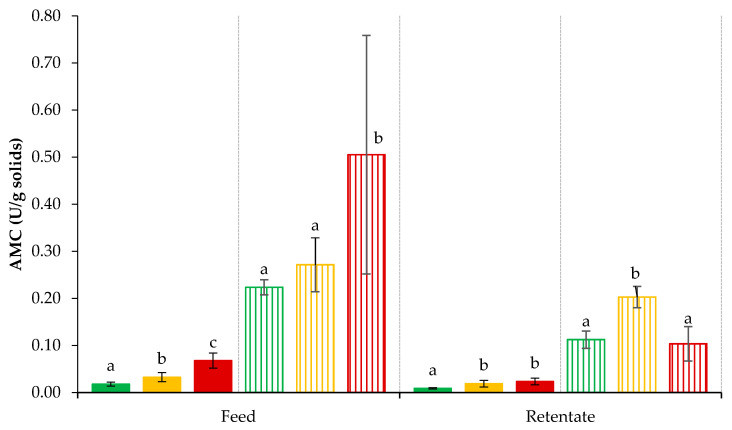
Innate plasmin (PL)- and plasminogen (PG)-derived plasmin activity in feed and UF retentate obtained at 5 ± 0.5, 20 ± 0.5, or 50 ± 0.5 °C. Bar colour represents PL at 5 °C (■), PL at 20 °C (■), PL at 50 °C (■), PG at 5 °C (║), PG at 20 °C (║), PG at 50 °C (║). Values are the raw mean ± standard deviation of at least duplicate analysis from triplicate runs at each test temperature. Different lower-case alphabet letters within feed PL and PG and retentate PL and PG indicate significant differences (*p* < 0.05).

The innate plasmin activity of feed and retentate samples generated at each processing temperature is shown in Figure 6. It is expressed as AMC units/g solids for the purpose of comparing plasmin activity between feed and concentrated retentate. Among the feed samples, the plasmin activity of feed processed at 50 °C was higher than that processed at 5 or 20 °C. The starting feed used in each UF run was equilibrated at processing temperatures of 5, 20, and 50 °C for 30 min, before collecting the samples for enzyme activity analysis. In milk, the concentration of inactive plasminogen is 2 to 30 times higher than plasmin. The equilibration of feed at 50 °C can cause denaturation of inactive plasminogen, as its denaturation temperature ranges between 50.1 °C and 61.6 °C [12]. Once denatured, plasminogen loses its naturally occurring tertiary structure and becomes more accessible to the action of heat stable plasminogen activator, which can convert inactive plasminogen into active plasmin, contributing to higher plasmin activity, as observed in feed F50 [39]. When compared to feed samples, all retentate samples had lower plasmin activity. The UF membranes typically used for concentrating milk proteins range in molecular weight cut-off from 5–15 kDa, which is much smaller than the size of plasmin and plasminogen at ≈48 and ≈88 kDa, respectively, as well as its inhibitors, plasminogen activator inhibitor (55 kDa) and plasmin inhibitor (60 kDa) [12,13]. Therefore, the complete retention of plasmin and other components of the plasmin system can be expected during UF. The retention of inhibitors with UF concentration could have contributed to the higher overall inhibition of plasmin. Subsequently, the inhibition by plasmin system inhibitors could have influenced the overall plasmin activity in R5, R20, and R50. Moreover, the whey proteins β-LG and α-LA are known to have strong inhibitory action against plasmin, which could suppress plasmin activity in high protein systems [40]. Therefore, the increase in concentration of proteins with UF, particularly whey proteins, must also be taken into consideration to interpret the resulting lower plasmin activity of retentate as compared to feed when measured on the basis of total solids (AMC/g solids). The plasmin activity also varied significantly (*p* < 0.05) among retentate samples, with R5 displaying lower activity compared to R20 and R50. As described earlier, the role of plasminogen activator in converting inactive plasminogen to plasmin is noteworthy in explaining the difference in plasmin activity between retentate samples. The duration required for temperature equilibration in starting feed batch and subsequent UF processing at 5, 20, and 50 °C could have influenced the action of plasminogen activator [12]. The UF of skim milk at 50 °C may have facilitated the denaturation of inactive plasminogen and its activation into plasmin by action of plasminogen activator [11], which may contribute in overall plasmin activity, as observed in R50. At 20 °C, plasminogen activation may take place because it is somewhat close to optimum activity (37 °C) temperature [12], which may increase plasmin activity, as seen in R20.

The plasminogen-derived activity was measured after incubating feed and retentate samples with added urokinase, a plasminogen activator, which can cause activation of inactive plasminogen to active plasmin, even when added at very low concentrations [41]. The urokinase-activated plasmin activity was up to 8–10 times higher in all samples when compared with innate plasmin activity (Figure 6). It suggests the presence of at least 8–10 times higher levels of inactive plasminogen in feed material, which was activated to plasmin on incubation with urokinase [12]. The occurrence of urokinase-activated plasmin activity in retentate samples suggests that inactive plasminogen must have been retained during UF. Consequently, its presence in retentate could contribute to proteolytic activity by conversion to plasmin, assisted by native plasminogen activator. UF retentate as an ingredient with low plasmin activity is interesting for multiple dairy applications such as cheese, high protein powders, and milks [14]. Therefore, strong inhibitory action of whey proteins and plasmin system inhibitors could be expected if they are extensively retained in retentates during UF of skim milk due to their relatively larger molecular size [42].

## 4. Conclusions

The results of this study show that UF of skim milk at 50 °C resulted in higher permeate flux than at 5 and 20 °C. However, the rate of flux decline during processing was considerably greater at this higher processing temperature, which was attributed to more extensive membrane fouling at 50 °C, as indicated by low permeability of clean water immediately post UF processing. These differences in fouling impacted protein profile of the resultant filtration streams and would also be expected to impact on cleaning-in-place of such membranes. The retentate produced at 5 °C had the highest viscosity. The plasmin activity in retentates increased in the order 5 < 20 < 50 °C. UF processing temperature significantly affected permeation of soluble minerals due to temperature-induced alterations mineral equilibria. These changes directly influence the physicochemical and functional properties of dairy ingredients derived from UF retentate and permeate streams.

## Figures and Tables

**Table 1:** Composition and physicochemical properties of retentates and permeates obtained by ultrafiltration of skim milk at 5 ± 0.5, 20 ± 0.5, or 50 ± 0.5 °C.

Parameter	Feed	UF Retentate			UF Permeate		
		5 °C	20 °C	50 °C	5 °C	20 °C	50 °C
TS (%, *w/w*)	8.44 ± 0.11	18.6 ^b^ ± 1.96	15.6 ^a^ ± 1.38	20.4 ^c^ ± 1.34	5.19 ^b^ ± 0.04	5.15 ^a^ ± 0.01	5.31 ^c^ ± 0.04
Protein (%, *w/w*)	3.03 ± 0.13	12.1 ^a^ ± 1.29	9.83 ^a^ ± 1.12	13.7 ^b^ ± 1.15	0.10 ^a^ ± 0.01	0.11 ^ab^ ± 0.01	0.12 ^b^ ± 0.01
Lactose (g/L)	58.7 ± 3.69	42.0 ^c^ ± 0.78	38.1 ^b^ ± 2.26	25.3 ^a^ ± 0.96	44.7 ^a^ ± 5.23	43.9 ^a^ ± 2.43	50.7 ^a^ ± 4.75
Ash (%, *w/w*)	0.62 ± 0.06	1.31 ^a^ ± 0.16	1.18 ^a^ ± 0.20	1.58 ^b^ ± 0.13	0.35 ^a^ ± 0.04	0.35 ^a^ ± 0.10	0.40 ^a^ ± 0.06
pH	6.75 ± 0.06	6.73 ^a^ ± 0.04	6.78 ^a^ ± 0.02	6.75 ^a^ ± 0.05	6.86 ^b^ ± 0.08	6.82 ^b^ ± 0.03	6.59 ^a^ ± 0.06
Ionic Ca^2+^ (mM)	2.22 ± 0.33	2.35 ^ab^ ± 0.40	3.00 ^b^ ± 0.44	2.20 ^a^ ± 0.15	2.53 ^b^ ± 0.51	3.43 ^b^ ± 0.59	1.57 ^a^ ± 0.01
Conductivity (mS/cm)	4.80 ± 0.05	3.57 ^a^ ± 0.37	4.01 ^a^ ± 0.18	3.71 ^a^ ± 0.18	5.09 ^a^ ± 0.11	5.14 ^a^ ± 0.05	5.15 ^a^ ± 0.06
		**Feed**			**UF Retentate**		
		5 °C	20 °C	50 °C	5 °C	20 °C	50 °C
Zeta potential (mV)		−26.4 ^b^ ± 3.25	−27.8 ^ab^ ± 0.88	−28.9 ^a^ ± 1.34	−26.6 ^a^ ± 1.19	−26.8 ^a^ ± 1.11	−26.5 ^a^ ± 1.01
Particle size (nm)		187 ^b^ ± 4.17	189 ^b^ ± 3.01	179 ^a^ ± 4.46	190 ^b^ ± 11.8	189 ^b^ ± 4.06	179 ^a^ ± 4.11

Values are the raw mean ± standard deviation of at least duplicate analysis from triplicate runs at each test temperature. Different superscripted lower-case alphabet letters within a row indicate significant differences (*p* < 0.05).

**Table 2:** Profile of individual minerals in retentates and permeates obtained by ultrafiltration of skim milk at 5 ± 0.5, 20 ± 0.5, or 50 ± 0.5 °C.

Minerals (mg/100 g)	Feed	UF Retentate			UF Permeate		
		5 °C	20 °C	50 °C	5 °C	20 °C	50 °C
Calcium	94.9 ± 4.9	319 ^a^ ± 33.5	271 ^a^ ± 39.9	395 ^b^ ± 22.7	27.9 ^b^ ± 1.0	26.0 ^b^ ± 1.8	20.9 ^a^ ± 1.3
Phosphorus	93.7 ± 4.5	257 ^ab^ ± 42.9	214 ^a^ ± 17.3	281 ^b^ ± 13.8	40.2 ^b^ ± 1.6	37.8 ^ab^ ± 1.4	35.3 ^a^ ± 1.2
Sodium	29.1 ± 0.1	32.0 ^b^ ± 0.7	30.1 ^a^ ± 0.3	32.3 ^b^ ± 0.9	28.0 ^a^ ± 1.0	28.0 ^a^ ± 0.4	28.3 ^a^ ± 2.1
Magnesium	9.3 ± 0.2	20.1 ^b^ ± 1.7	16.9 ^a^ ± 1.7	21.2 ^b^ ± 1.1	5.3 ^a^ ± 0.2	5.3 ^a^ ± 0.1	5.5 ^a^ ± 0.4
Potassium	137 ± 0.6	155 ^b^ ± 3.3	147 ^a^ ± 1.8	160 ^b^ ± 3.5	133 ^a^ ± 4.1	135 ^a^ ± 1.5	138 ^a^ ± 9.9
Sulphur	34.8 ± 1.8	106 ^ab^ ± 10.6	87.6 ^a^ ± 11	119 ^b^ ± 6.8	13.0 ^a^ ± 0.8	13.3 ^a^ ± 0.3	13.5 ^a^ ± 0.6
Iron	0.5 ± 0.7	0.1 ^a^ ± 0.1	0.1 ^a^ ± 0.1	0.1 ^a^ ± 0.0	0.1 ^a^ ± 0.0	0.1 ^b^ ± 0.1	0.0^a^ ± 0.0
Copper	0.0 ± 0.0	0.0 ^ab^ ± 0.0	0.0 ^a^ ± 0.0	0.0 ^b^ ± 0.0	0.0 ± 0.0	0.0 ± 0.0	0.0 ± 0.0
Zinc	0.3 ± 0.0	1.3 ^ab^ ± 0.2	1.1 ^a^ ± 0.1	1.5 ^b^ ± 0.1	0.0 ± 0.0	0.0 ± 0.0	0.0 ± 0.0
Ca: Phosphorus	1.0 ± 0.1	1.3 ^a^ ± 0.1	1.3 ^a^ ± 0.2	1.4 ^a^ ± 0.0	0.7 ^b^ ± 0.0	0.7 ^b^ ± 0.0	0.6 ^a^ ± 0.0
Ca: Protein (mg/g)	31.2 ± 1.6	26.4 ± 0.9	27.6 ± 1.5	28.9 ± 2.6	-	-	-

Values are the raw mean ± standard deviation of at least duplicate analysis from triplicate runs at each test temperature. Different alphabet letters for retentate and permeate samples within a row indicate significant differences (*p* < 0.05).
